# Open Up – the Mission Statement of the Control of Impulsive Action (Ctrl-ImpAct) Lab on Open Science

**DOI:** 10.5334/pb.494

**Published:** 2019-08-20

**Authors:** Christina B. Reimer, Zhang Chen, Carsten Bundt, Charlotte Eben, Raquel E. London, Sirarpi Vardanian

**Affiliations:** 1 Department of Experimental Psychology, Ghent University, BE

**Keywords:** Open Science, Open Access, Open Data, Open Methodology, Open Source, Transparency

## Abstract

The present paper is the mission statement of the Control of Impulsive Action (Ctrl-ImpAct) Lab regarding Open Science. As early-career researchers (ECRs) in the lab, we first state our personal motivation to conduct research based on the principles of Open Science. We then describe how we incorporate four specific Open Science practices (i.e., Open Methodology, Open Data, Open Source, and Open Access) into our scientific workflow. In more detail, we explain how Open Science practices are embedded into the so-called ‘co-pilot’ system in our lab. The ‘co-pilot’ researcher is involved in all tasks of the ‘pilot’ researcher, that is designing a study, double-checking experimental and data analysis scripts, as well as writing the manuscript. The lab has set up this co-pilot system to increase transparency, reduce potential errors that could occur during the entire workflow, and to intensify collaborations between lab members. Finally, we discuss potential solutions for general problems that could arise when practicing Open Science.

Working in science is fulfilling, but can also be frustrating at times. As early-career researchers (ECRs), we get frustrated when we try to download an interesting paper, but hit a paywall; when we plan to use an existing paradigm, but cannot exactly reproduce it as the methods information is incomplete and the materials and code are unavailable; when we want to try out a new analysis on a data set, but cannot get access to it; and when our paper gets rejected, because the results are not statistically significant and are thus deemed unpublishable. Incidents like these made us reflect on how science should be done and why we want to be scientists. In this paper, we will share our thoughts and answers on these questions that we found within the practices and philosophy of Open Science.

Since October 2018, we are working together in the Control of Impulsive Action (Ctrl-ImpAct) lab, led by Frederick Verbruggen at Ghent University (https://fredvbrug.github.io). Our principal investigator (PI) Frederick Verbruggen has been practicing Open Science for some years now, which is why interest and/or experience in Open Science is considered a prerequisite for joining the lab. As a lab, we are all convinced that we should base our research routine on the principles of Open Science, “the practice of science in such a way that others can collaborate and contribute, where research data, lab notes and other research processes are freely available, under terms that enable reuse, redistribution and reproduction of the research and its underlying data and methods” (Foster, n.d.).

Open Science applies to all research disciplines. Here, we will address the topic from the perspective of a cognitive psychology and neuroscience lab. More specifically, the present paper is our lab’s mission statement regarding Open Science. The paper consists of three parts. First, we as ECRs would like to contribute to the discussion on Open Science by outlining our personal motivation for Open Science; second, we will describe how we incorporate Open Science practices into our scientific workflow; and third, we will discuss potential solutions for general problems that could arise when adopting Open Science practices. This mission statement is our lab’s public commitment to Open Science. We hope it will also inspire and help other labs and/or researchers to base their research routine on the principles of Open Science, by providing a concrete example of how they can incorporate Open Science practices into their own scientific workflow.

We are aware that many paths lead to a common goal, and that the Open Science practices we adopt may not necessarily suit everyone. Furthermore, with the emergence of new ideas and technologies, good research practices of today may be deemed inappropriate tomorrow, and better ways of conducting research may appear. Our mission statement thus reflects what seems most useful to us at the present time, and we are aware that for other researchers or in other times, ideas about what good research practices entail may change. We will therefore continuously reflect on how we do research and how we can further improve our workflow. The specific guidelines that we develop in the lab will be shared on GitHub (see links in the Appendix), and we invite other researchers to participate by for instance providing feedback and sharing experiences. With this paper, we thus also wish to reach out to other researchers and start an ongoing exchange on how we want to do research as a community.

## Our personal motivation to follow Open Science

To start thinking about why we as ECRs embrace Open Science, we first need to rethink what science is, why we as ECRs want to pursue an academic career, and what we consider as the output of scientific research. For many, the output of one’s research may be the publication that summarizes the findings. However, as John Claerbout, an earth scientist at Stanford, put it, “an article about a computational result is advertising, not scholarship. The actual scholarship is the full software environment, code and data, that produced the result.” (as quoted in Donoho, 2010, p. 385). In psychology research, one may similarly argue that journal articles are just part of, but not the whole scholarship. The whole scholarship lies not only in the motivating research question and theoretical background (which is usually part of journal articles), but also in study materials and experimental scripts, collected data, and data analysis scripts used to produce the results. In other words, all products generated in a research project should be considered research output. This view dovetails well with what distinguishes scientific findings from mere opinions and hearsay. In many fields, including our own, science is based on empirical testing and the reproducibility of empirical findings, rather than mere trust between individual scientists (Klein et al., 2018). When a finding is shared, the scientific community needs to carefully assess and critically evaluate the accuracy of the finding, independently replicating the results multiple times, before it becomes accepted as part of scientific knowledge. However, when the study materials, data, experimental and analysis scripts supporting the reported finding are not available, it becomes difficult or sometimes even impossible for scientists to verify it. Such unverifiable findings cannot be considered as scientific findings. Open Science, in our opinion, is thus just what science entails, whereas siloed science is not science.

In addition to our strong conviction that Open Science is just science done right (Imming & Tennant, 2018), following the principles of Open Science also confers multiple benefits (compared to science practiced in a closed manner). Transparency is at the heart of Open Science, and we believe that transparent workflows increase the credibility of the research. When products of all stages of research (i.e., study plans, raw data, experimental and analysis scripts, study materials, publications) are available, researchers enable their colleagues and anyone who is interested to completely retrace their work and build on it. The possibility to examine all steps that precede a publication will likely increase the credibility of the findings, because all relevant information is available for close scrutiny, not only the summarized version in form of publication. Such rich information would enhance the understanding of each other’s work and inspire new ideas (Colavizza, Hrynaszkiewicz, Staden, Whitaker, & McGillivray, 2019). Moreover, by making study materials and experimental scripts open, researchers can more easily replicate others’ work. As argued above, we consider replicability and thus replication of empirical findings an essential part of science. By increasing the reputation of replication studies and making replication studies easier to conduct, Open Science would motivate more researchers to invest time and resources into such investigations, which may eventually increase the overall credibility of the psychological literature.

From a pragmatic point of view, we are convinced that Open Science helps avoid effort duplication and allows ECRs like us to get a smoother start into new research projects. Having access to materials of previous experiments can provide valuable opportunities for us to improve our research skills, especially at the beginning of new projects. Programming new experiments by using functioning scripts saves time and could reveal important design choices that we would miss otherwise. Especially when programming skills are still low, improving these skills is easier when starting with working examples than from scratch. This may especially be the case for ECRs who cannot fall back on a collection of scripts and study materials yet. In addition, having access to data and data analysis scripts of the studies we want to build on gives us the chance to reanalyze the data or try new analyses before building our own experiments on them (Leonelli, 2018).

Apart from what in our opinion are good scientific practices, we would like to emphasize that Open Science offers researchers much more. Open Science can foster creativity. In today’s publication culture, we notice that many researchers – including ourselves – seem to have a strong focus on positive findings (i.e., statistically significant findings) when designing new studies. For example, some researchers seem to make tidy but only small variations to familiar questions and established paradigms, instead of addressing new open questions and designing new paradigms. Thus, these researchers seem to follow an approach that is more likely to show positive results. This mindset is understandable, given a culture where a publication is seen as the sole research output and where positive results are deemed more publishable than null results. In our opinion, this mindset is restricting and limits researchers’ thinking in a way that hinders the advance of science. Open Science has the potential to liberate researchers from this restrictive mindset in at least two ways. First, when one thinks of all products from a scientific project as research output, the fixation on the final results and the publication will necessarily decrease. Carefully created study materials, novel experimental tasks, and sophisticated data analysis methods are all valid and useful output from one’s research, regardless of whether the final result is positive or not. By thinking of and evaluating research output in such a broad sense, researchers will not need to forsake creativity for positive findings. Doing research may also become more fulfilling and rewarding, as each line of code one writes, and each data set one collects may become small building blocks that eventually make up the edifice of solid science. Second, certain Open Science practices will help researchers to adopt a creative mindset when thinking about new studies (Allen & Mehler, 2019; Frankenhuis & Nettle, 2018). This is mainly accomplished by pre-registering studies, as pre-registration allows for evaluating study quality instead of the significance of the findings (Nosek, Ebersole, DeHaven, & Mellor, 2018). Researchers can pre-register studies by themselves on platforms such as the Open Science Framework (OSF; osf.io) or submit their study plans to journals as registered reports (Chambers, 2013; Hardwicke & Ioannidis, 2018). In the latter case, a study plan is peer-reviewed before the study is conducted. If the study plan is approved and the study is well conducted, it will be published irrespective of whether the findings are positive or null. Especially for us as ECRs, registered reports offer a chance to integrate reviewer comments in the study plan before data collection and this could help us design better studies and conduct higher quality research. All in all, Open Science practices such as pre-registrations empower researchers to follow their curiosity irrespective of the outcome of the research, by changing the main criterion for publication from merely positive findings towards high quality research. This, in turn, may help researchers daring to leave familiar paths and start exploring unfamiliar terrain, starting new collaborations and fostering novel and creative approaches to important theoretical questions.

Lastly, we consider doing science in an open manner an ethical obligation. As researchers working at a public university, with research funded by taxpayer’s money, we feel it is imperative to make our research open to the public. Although as we argued above, all products from a research project should ideally be made open, Open Access publication is of special relevance here, as the published article has potentially the largest audience. Furthermore, having access to an article is the first step towards discovering the corresponding study materials, scripts, and data. As employees of Ghent University, we have access to the majority of journals in our field. However, this is not the case for other – and especially non-Western – universities and research institutes, and also not for the general public who is not affiliated with universities and research institutes. One major motivation for us to work in science is to contribute to collective human knowledge, and we would like our contribution to be accessible for as many people as possible. When publications are freely available, they are more likely to be read and cited (Piwowar & Vision, 2013; Wang, Liu, Mao, & Fang, 2015). In that sense we believe that Open Access publications could increase the diversity of the scientific community, inspire new research, improve the scientific progress and help to spend public money more thoughtfully (McKiernan et al., 2016).

Despite our conviction that Open Science confers multiple benefits, we do acknowledge that this endeavor may have potential downsides. We will discuss some of them in more detail throughout and especially in the last part of the paper. Here we want to mention two potential risks that researchers, especially ECRs, should be aware of when following Open Science (Allen & Mehler, 2019). First, integrating Open Science practices into the research routine can be time-consuming at the beginning. The time spent writing pre-registrations, anonymizing raw data files and sharing experimental and data analysis scripts means that less time is available for other tasks. Second, Open Access publications could mean publications in journals with lower impact factors, so that Open Science practitioners could appear less competitive on their CVs. At first glance, this may sound like bad news, but it should not scare researchers away from considering the move to Open Science. In the following, we will describe the Open Science practices in our lab in more detail and argue why we think the benefits of Open Science outweigh these potential downsides.

## The Open Science workflow in our lab

**Figure 1:**
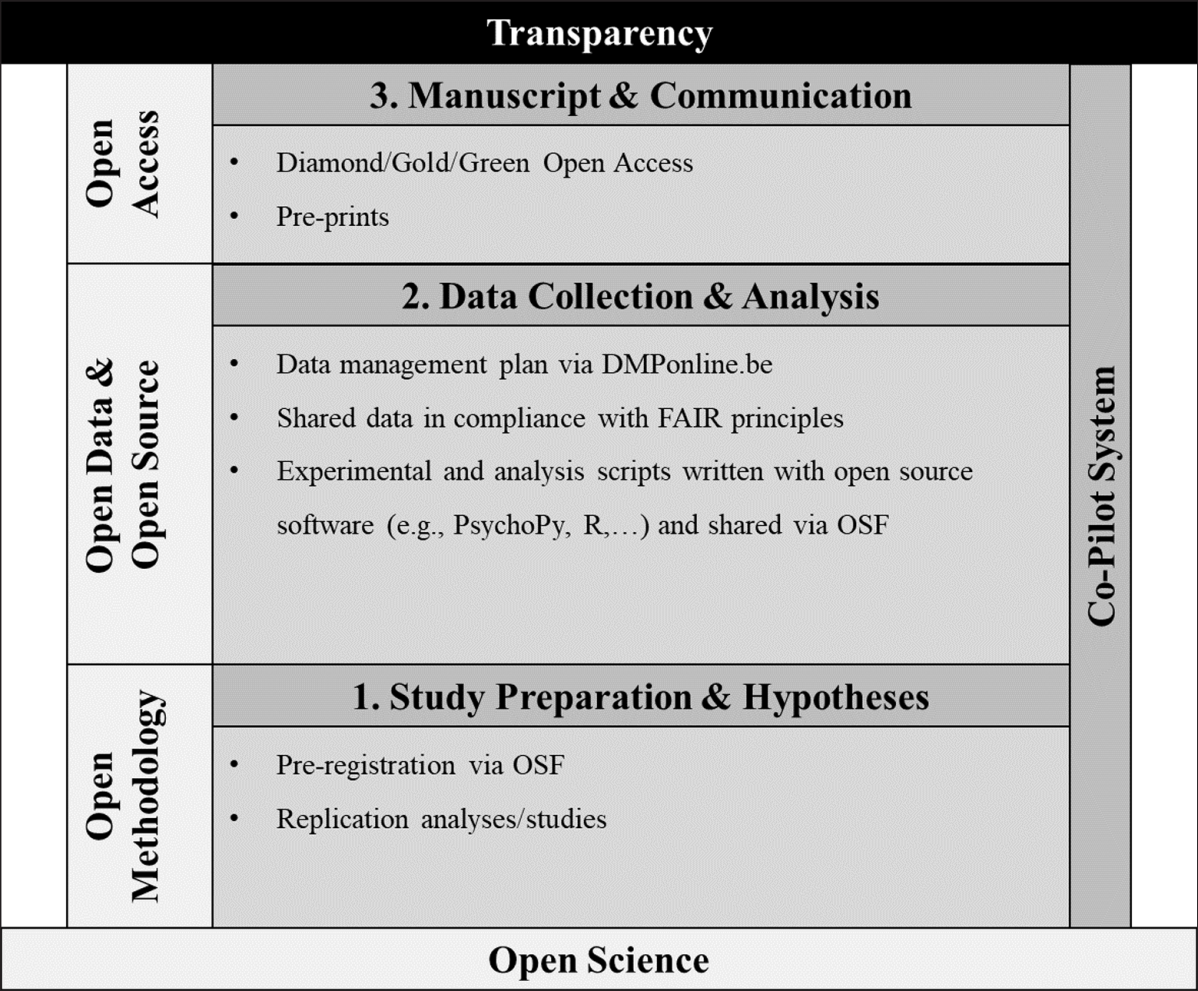
The figure illustrates how the Control of Impulsive Action (Ctrl-ImpAct) Lab will implement the principles of Open Science in order to contribute to a replicable and transparent scientific practice. In our perspective, the main goal of Open Science is to increase scientific transparency, and this can be achieved by the implementation of well-defined steps throughout the scientific workflow. Importantly, in the Ctrl-ImpAct Lab, the implementation of a co-pilot system will ensure quality of the entire workflow.

Open Science is an umbrella term that encompasses multiple aspects of a research process. In our mission statement, we will focus on four aspects of Open Science that are particularly relevant for our lab: Open Methodology, Open Data, Open Source, and Open Access. As can be seen in Figure 1, these Open Science practices are embedded in our ‘co-pilot’ system, in which a lab member is involved in another lab member’s project from the very beginning until the end to check each step in the whole research process. In the following, we will first outline the ‘co-pilot’ system, and then describe how we incorporate the Open Science practices into our scientific workflow as a lab.

## Co-Pilot system

Being human, making mistakes is almost inevitable. To reduce potential errors and increase the reliability of scientific knowledge, the scientific community has implemented several quality control mechanisms, such as the collaboration of multiple authors on a paper to double-check each other’s work, and the expert peer-review procedure that papers need to go through before publication. However, it seems that not every step in the research process has received similar levels of scrutiny. For instance, a survey with psychologists revealed that double-checking each other’s data analysis and reported results was uncommon (Veldkamp, Nuijten, Dominguez-Alvarez, van Assen, & Wicherts, 2014). Accordingly, reporting errors in statistical results in psychology papers are relatively prevalent (Bakker & Wicherts, 2011; Nuijten, Hartgerink, van Assen, Epskamp, & Wicherts, 2016). To reduce such errors, a quality control system that checks each step in the research process is needed.

In working environments where human errors are common and may have huge consequences, systems have been implemented to help reduce errors (Reason, 2000). One such system can be found in aviation, namely the co-piloting system, where the co-pilot double-checks every step of the pilot. The co-piloting system seems to significantly reduce the risk of human errors leading to airplane crashes (Wiegmann & Shappell, 2003).

Wicherts (2011) argued that scientists should learn from how aviation deals with human errors. He proposed the ‘co-pilot’ system as a way to reduce errors in statistical analysis, in which at least two researchers conduct statistical analyses independently to increase the chance of veracity of reported results. We believe that such a co-pilot system should not be limited to data analysis, but should include other steps in a research process as well. That is why our lab implements a similar ‘co-pilot’ system. For each study, there is a lead researcher (or ‘pilot’) and a ‘co-pilot’, who is involved in the entire workflow. The ‘co-pilot’ researcher not only double-checks the data analysis scripts of the ‘pilot’ researcher, but is also involved in other tasks, from the conception of a research idea until the publication of the manuscript.

We are using the co-pilot system since February/March 2019. We implemented the co-pilot system via GitHub, as GitHub allows for version control and provides integration with OSF that we use for pre-registering experiments and sharing data, scripts, and study materials (see below). At the beginning of a new project of a lab member (the pilot researcher), another lab member will be assigned as his/her co-pilot. The pilot researcher creates a private GitHub repository for the project and invites the co-pilot as a collaborator. In this way, the co-pilot has full access to all materials used in the project. Pre-registrations, experimental scripts, anonymized data files, and data analysis scripts are shared on GitHub between the pilot researcher and the co-pilot. The co-pilot then checks the work of the pilot researcher at each step. For instance, the co-pilot scrutinizes the experiment documentation (or pre-registration) to ensure that the research question, hypothesis, and data analysis plan are accurate and as intended. The co-pilot also double-checks the experimental scripts by running the experiment on him or herself and by working through the code. In the case of data analysis, the co-pilot starts with the anonymized raw data files, and checks the analysis code step by step. The co-pilot can also analyze the data independently or try out alternative data analysis techniques to see if the results obtained by the pilot researcher are robust (Steegen, Tuerlinckx, Gelman, & Vanpaemel, 2016). In each instance, the co-pilot provides feedback via GitHub, and the pilot researcher incorporates the comments from the co-pilot. A checklist for all the steps involved in the co-pilot system and more detailed guidelines can be found in the Appendix.

The co-pilot system will not only increase the quality of our work by weeding out potential errors, it may also make our work more impactful by allowing other researchers to build on it more easily. For the co-pilot system to work well, the pilot researcher needs to provide thorough documentation for experimental scripts, raw data, and data analysis scripts. By making these materials open, we are thus creating and sharing sustainable knowledge, which allows our future selves and other researchers to easily reuse the data, adapt the experimental scripts, and reproduce the analyses (Hardwicke et al., 2018; Leonelli, 2018; Simmons, Nelson, & Simonsohn, 2011; Stodden, Seiler, & Ma, 2018). Our work will be useful not only in terms of publication, but also in all other research outputs that we generate throughout the project.

We are aware that running such a co-pilot system is costly in time. After all, each of us needs to not only work on our own projects, but also be co-pilots for others. Moreover, writing comprehensive documentation is also time-consuming. This investment in time may well slow down the progress in our lab. One way to address this problem is to reward co-pilots with meaningful co-authorships. Contributions of each co-author will thus be clearly and transparently reported using the CRediT taxonomy (Brand, Allen, Altman, Hlava, & Scott, 2015), such that the contributions of each author, including that of the co-pilot, will be clear. Furthermore, writing documentations may be time-consuming in the short-term, but in the long-term it may save us time, as we will not need to retrospectively prepare data files or scripts when other researchers request them. By making these materials open, other researchers can reuse them without our intervention (although, of course, we would love to hear from other researchers who are using our data, experimental and data analysis scripts or study materials). Furthermore, by focusing on quality rather than quantity (The Slow Science Academy, 2010), we may eventually save time for both our lab and the whole scientific community by reducing errors in our findings.

So far, the co-pilot system is working well in our lab. We try to distribute the workload evenly and discuss regularly whether the workload is acceptable. It has not been a problem yet to assign co-pilots, since we all have similar skills that are required to check each other’s work. Moreover, the close collaboration among lab members enables us to learn from each other and thus to improve and align our skill sets. However, this situation may change, for example when some of us move on to specific and/or more advanced analysis techniques or methodologies. This situation has in fact already occurred. When none of us has the required skills to qualify as co-pilot, we ask researchers with the required expertise outside the lab to collaborate and act as co-pilots. One project may thus require multiple co-pilots, each focusing on a specific aspect of the project.

In our view, science is an inherently collaborative enterprise. Implementing the co-pilot system fosters close collaboration among lab members, which is the way we would like to work. Furthermore, sharing one’s finding with the whole scientific community is exciting, but can also be nerve-racking for ECRs, as one can never be 100% certain that no error is made along this complicated and at times daunting process. Having someone closely check each step of your work helps alleviate this feeling of uncertainty. Being involved in each other’s projects also allows us to learn Open Science practices from each other, which will be important assets in our career. In the following sections, we will describe the specific Open Science practices in more detail.

## Open Methodology

Some have argued that psychological science is in the middle of a replication crisis, in which many previously reported findings – including several highly cited and influential ones – cannot be replicated on subsequent investigation (Camerer et al., 2018; Ebersole, Axt, & Nosek, 2016; Open Science Collaboration, 2015; R. A. Klein et al., 2014). This lack of replicability has severely reduced confidence in the published literature among psychological scientists (Pashler & Wagenmakers, 2012). For ECRs who wish to devote themselves to and have an impact on the field, this situation can be especially disheartening, as the findings they wish to build on may turn out to be unreliable.

Many questionable research and publication practices may have contributed to the current replication crisis. Among them, the most prominent causes include: low statistical power (Button et al., 2013), flexibility in data collection and analysis, selective reporting (Simmons et al., 2011), presenting unexpected findings as having been a priori predicted (or HARKing, hypothesizing after results are known; Kerr, 1998), and publication bias of favoring novel positive findings over null results (John, Loewenstein, & Prelec, 2012). Researchers may engage in such questionable practices either intentionally or unintentionally to generate publishable positive findings (Nuzzo, 2015). It is beyond the scope of the current paper to discuss in detail the prevalence of these questionable practices in the field and how each of them may have contributed to the replication crisis. It should be sufficient to say that all these questionable research and publication practices lead to the proliferation of Type I errors or overestimation of effect sizes in the published literature in one way or another. When researchers try to reproduce previous findings, they often fail, as the original finding is more often than we would like a false positive or an overestimation that is ushered into the literature by these questionable practices.

Although not everyone agrees that (psychological) science is facing a replication crisis (Fanelli, 2018; Gilbert, King, Pettigrew, & Wilson, 2016; Stroebe & Strack, 2014), we think science will benefit from adopting improved and more transparent research practices, regardless of whether there is a crisis or not (Fanelli, 2018). We thus do not need the replication crisis to embrace Open Science.

One Open Science practice that helps address the above-mentioned questionable research practices is pre-registration (Nosek et al., 2018). In the Ctrl-ImpAct lab, we will make study pre-registration a core part of Open Methodology and will routinely pre-register our studies, either via OSF or via registered reports. In a pre-registration, we will specify (1) the motivating research question and hypothesis, (2) the research design and study materials including planned sample size, (3) the outcome variables, and (4) the predictor variables and a more specific data analysis plan, before data collection has started.

Each of the components in a pre-registration helps safeguard against certain questionable research practices. For example, pre-registering one’s motivating research question and hypothesis makes it less likely to report unexpected findings as having been predicted a priori. (As a reviewer pointed out, technically it is still possible to cheat by ‘pre-registering’ a study after data collection is finished. However, we believe most researchers would consider this behavior as outright fraud and would not engage in it.) Specifying the sample size of a study in advance eliminates the possibility of selective stopping during data collection. Although pre-registration may not necessarily solve the problem of insufficient statistical power, as one can still pre-register studies with low power, we think that it will still be helpful in alleviating the problem. Having to decide upon and pre-register a certain sample size forces one to carefully think about one’s choice and justification for the choice (which is often provided by formal power analysis), rather than simply rely on inappropriate rules of thumb. Pre-registering research design and study materials prevents selective reporting, as one may not drop conditions and variables that do not ‘work’. Lastly, pre-registering the predictor variables and the outcome variables, or a more specific data analysis plan reduces the flexibility in data analysis, so that a researcher can no longer try out all kinds of analyses and just report the one that gives the ‘best’ result. Together, pre-registration helps scientists guard against the questionable research practices mentioned above.

Pre-registration of confirmatory research will be a given in our lab. In the case of exploratory research, we will not be able to specify all components of a pre-registration in advance. In some cases, we think pre-registration can still be useful, when some aspects of exploratory research can be specified in advance (general theoretical background and assumptions, sample size, or certain data analysis choices). Pre-registration of exploratory research will safeguard against our own hindsight bias in such cases. Furthermore, both confirmatory and exploratory studies will be shared on OSF. OSF will thus contain a record of all studies that our lab has conducted.

All pre-registrations will be reviewed within our lab before the planned study is executed (see the Co-Pilot system above). In some cases, where the research question can be addressed by a single well-designed study, we may send out the pre-registration to expert reviewers in the form of registered reports (Chambers, 2013; Hardwicke & Ioannidis, 2018). Many journals nowadays accept registered reports (for a list of such journals, see https://cos.io/rr/), in which a study plan is reviewed and in principle accepted before data collection has started. After executing the study as planned, the paper will be published regardless of its results, thus providing a way to reduce publication bias. For other pre-registered studies that are not published as registered reports, we will write up the results and submit to journals regardless of whether the results are positive or not. In our opinion, provided that studies have been conducted well, every outcome is a gain of knowledge. In the spirit of Open Science, both positive and negative findings should be shared. In some cases, it may not be possible to publish our findings (positive or negative) via regular journal articles. For instance, sometimes we may try out a new paradigm that turns out to be unsuitable, or we may decide to use a different procedure after trying out a certain procedure. In such cases, the contribution of the studies may be too incremental to be published in scientific journals. However, sometimes sharing these half-baked ideas or dead-ends can also be important, as they may inspire other researchers or help them save time and money by not going down the same path. When we believe that such studies could be informative for others, we will write up the results and share them as pre-prints to make them available to the scientific community. We will carefully select which studies we will share and review the documents we upload within our lab to guarantee the quality, so that pre-print servers will not be overloaded with pre-prints of low quality.

When the pre-registered studies are published in the form of registered reports, regular journal articles or pre-prints, we will make the pre-registrations public and provide links to them in the manuscripts. Furthermore, links to the manuscripts will also be provided on OSF for each public project. The correspondence between a pre-registration and the eventual reported study would become clear: it will be easy to check whether a certain reported study is pre-registered or not, and whether the results of a pre-registered study have been shared.

Pre-registration reduces questionable research practices by tying researchers’ hands in advance when they pre-commit to a certain analysis and reporting plan (Nuzzo, 2015). Some researchers are thus concerned that pre-registrations do not allow for changing plans and exploring data (van ’t Veer & Giner-Sorolla, 2016; Washburn et al., 2018). However, we think this may be a misconception, as deviations from pre-registrations can occur and should not be penalized (Frankenhuis & Nettle, 2018; Hardwicke et al., 2018). When deviations from pre-registrations occur, we will report these deviations in a transparent manner in the manuscript. The same holds for exploratory data analysis. If results are obtained with exploratory analysis, we will report them as such. Furthermore, results of such exploratory analyses can lead to further confirmatory research, in which the study design and data analysis plan can be fully specified in advance.

Another concern with pre-registration is that writing pre-registrations requires more work. We think that this is not necessarily the case, as there are templates available that make pre-registrations easy (Houtkoop et al., 2018; Klein et al., 2018). Furthermore, researchers often need to provide detailed information for ethics boards to obtain ethics approval. Turning the documents for ethics boards into pre-registrations (or the other way around) may not take much extra work. In addition, the pre-registrations could be used as first drafts for the introduction and method sections of the manuscripts later on. Moreover, it may even save time, as planning ahead can avoid a lot of unnecessary mistakes. When working with high dimensional data such as electrophysiological or fMRI data, it can help constrain the analysis space ahead of time.

Lastly, some researchers are afraid of being scooped, which is why they refrain from pre-registering their studies (Houtkoop et al., 2018). However, such situations can easily be avoided. For instance, OSF provides an embargo period for up to four years, so that pre-registrations can be kept private until the pre-registered studies are published. Accordingly, we think that researchers do not need to worry that other researchers may scoop their ideas, as it is possible to make the pre-registrations public only after journals accept the manuscripts for publication. For peer-review, however, such private pre-registrations can be shared with reviewers by creating view-only links, so that the reviewers get all relevant information.

## Open Data and Open Source

As already mentioned, we consider examining the reproducibility of empirical findings as an essential part of scientific practice. The reproducibility of empirical findings can be examined on two different levels. One possibility is to independently reanalyze existing data of previous experiments, either with or without the corresponding analysis scripts, to see whether the reported findings can be reproduced (also referred to as *computational or analytical reproducibility*). Another possibility is to re-do previous experiments, and check whether similar findings can be obtained with new data (also referred to as *replicability*) (Leonelli, 2018; Patil, Peng, & Leek, 2016). Analytical reproducibility and replicability are two related but distinct concepts. We are convinced that examinations of reproducibility on both levels are necessary, since it is important for us to build our work on valid and reliable findings and to provide valid and reliable knowledge for other researchers to build on (Hardwicke & Ioannidis, 2018; Klein et al., 2018; Steegen et al., 2016; Stodden et al., 2018). Open Science could foster a cultural change so that the examination of reproducibility would be integrated into the scientific workflow (Giner-Sorolla, 2012; Ioannidis, 2005; Johnson, Payne, Wang, Asher, & Mandal, 2017; Pashler & Wagenmakers, 2012; Poldrack, 2019; Simmons et al., 2011). Such cultural change can be facilitated for example by opening journals for replication studies and by rewarding researchers for making their data and materials open so that others can more easily reproduce and replicate their findings (Gernsbacher, 2018; Kidwell et al., 2016).

To facilitate the examination of reproducibility, Open Data and Open Source are two important principles of our research routine. In practice, Open Data means for us that before starting a project, we will create a Data Management Plan (e.g., using DMPonline.be), because such plans facilitate data sharing (Houtkoop et al., 2018). As a general rule in our lab, data should be as open as possible, but as closed as necessary (see the link to our lab’s data management guidelines in the Appendix). This means that while only fully anonymized and non-sensitive data will be shared, highly confidential data will be shared under specific conditions (Lewandowsky & Bishop, 2016). In addition, we will make data compatible with the *FAIR principles*, which state that data of completed projects should be *F*indable, *A*ccessible, *I*nteroperable, and *R*eusable (Wilkinson et al., 2016). In more detail, data should be findable in a sense that they have sufficient metadata as well as a unique and persistent identifier. Data and metadata should be understandable and deposited in a trusted repository from where they are accessible. Moreover, metadata should be written in a formal and applicable language in order to make data interoperable. In other words, metadata should consist of clear and understandable vocabularies and they should include adequate references to other metadata. At the moment, data documentation seems insufficient in psychology. However, tools that help researchers make more standardized data documentation in an efficient way do exist (Arslan, 2019), and we will explore and incorporate such tools into our workflow. Lastly, data should have clear usage licenses that make them reusable. When projects are completed, we will make the fully anonymized and non-sensitive data publicly available on OSF or other trusted data repositories like university servers that provide long-term central infrastructure under a CC BY 4.0 license. We will also share an experiment documentation containing all information required to interpret the data files. Together with the analysis scripts that we will share, everyone should be able to move from the raw data to the reported results.

Regarding confidential data, the FAIR principles advise to publish rich metadata, so that the data themselves are easy to find. As mentioned above, we will use OSF to share such metadata. The metadata will contain rules specifying the processes and conditions for accessing the data. That is, everyone will be able to check if the data exist or not, but access will only be granted to persons who qualify. In case of very sensitive data or when misuse and abuse may be expected, we will ask an independent body, for example the ethics board of Ghent University, to arbitrate and decide who gets access.

We will also share experimental and analysis scripts as well as study materials. We aim at writing all new scripts in open source software, so that they are free to use for everybody. Proprietary software may still be used when no open source alternatives exist or when translating existing scripts in proprietary software to open source software is too time-consuming (for a discussion, see Wessel, Gorgolewski, & Bellec, 2019). In general, we strive to use open source software for all scripts. In our lab, experimental scripts are coded in PsychoPy (Peirce, 2007) or jsPsych (De Leeuw, 2015) and analysis scripts in R (R Core Team, 2013). To ensure long-term preservation of the scripts and materials, we will share them on OSF or other trusted repositories. To encourage the reuse of scripts and materials by other researchers, experimental and analysis code and other specialized software will be published under the GNU General Public License (GPL) 3.0, and study materials that we develop will be shared under a CC BY 4.0 license.

## Open Access

Open Access is another key principle of Open Science. Open Access literature is “digital, online, free of charge, and free of most copyright and licensing restrictions” (Suber, 2012, p. 4). We strongly aim for Open Access publications, as we believe that knowledge and in that sense education should be freely available for everyone. Therefore, we will strive for Diamond or Fair Gold Open Access. Diamond Open Access is “a publishing model that provides free access to peer-reviewed journal articles without charging […] [article processing charges] (APCs). Peer review and editing is done by volunteers” (Open Access Library, 2018a). On the other hand, Gold Open Access is “a publishing model that ensures free access to peer-reviewed articles through […] APCs” (Open Access Library, 2018b). Accordingly, we aim for publishing our work in fair/nonprofit Open Access journals that provide immediate Open Access to the articles and that require no APCs (Diamond) or low APCs (Gold). On our lab homepage, we will summarize the Open Access status of our publications (see https://fredvbrug.github.io/publications.html). Importantly, Diamond and Fair Gold Open Access will allow us to keep the copyright of our papers. This policy is also in line with the recommendation for Open Access publications of the ERC (European Research Council), the main funder of our lab, and of cOAlition S, a group of national research funding organizations (cOAlition S, 2019).

We will publish via Green Open Access (which is also supported by the ERC) only in case that Diamond or Gold Open Access is not possible or when there are no suitable alternatives for the specific studies (but our first choice will always be Diamond or Fair Gold Open Access). Green Open Access is also known as self-archiving, meaning that papers (usually post-prints) are published in an open repository after having been published in closed access journals for a specific embargo period. In such cases, preferences will be given to journals with no embargo periods.

Pre-prints should also be mentioned, as they seem to complement traditional publication routes. Pre-prints are manuscripts that are shared via repositories (e.g., psyarxiv.com) without having gone through peer-review. One should keep in mind, however, that not all journals accept manuscripts that have been previously made available as pre-prints (on sherpa.ac.uk/romeo, one can check journal policies concerning pre-prints). As a lab, we may consider pre-prints as well. Since we pre-register our study plans (see above) and plan to publish via Diamond or Gold Open Access, making our manuscripts available before submitting them to journals seems logical to us.

## General problems when practicing Open Science and adequate solutions

There are, of course, some obstacles on the road towards the adoption of Open Science practices. In the following, we will address a few general problems and provide solutions from our lab’s perspective.

First, advocating and implementing Open Science could invite criticism from researchers who may find errors in our work. If other researchers find errors in our work, we will transparently communicate the corrections. Although we believe that committing errors is human, being criticized by other researchers can still be difficult to handle. This may especially be the case for us as ECRs, since we may feel insecure about our qualification as researchers in such situations. Facing criticism together with all lab members, including our PI, gives us social support that makes it easier to take criticism. This in turn gives us the ability to use the criticism to our advantage and build on it. Taking criticism and building on it is a personal skill that can be acquired. Fostering an environment where it is safe to expose ourselves to criticism and where failure is an accepted part of work is thus crucial in this respect.

As a reviewer pointed out, many PIs or senior researchers do believe in Open Science, but may not recommend it to ECRs, because of possible drawbacks like lower productivity and publications with lower impact factors. This may be a legitimate concern, as researchers are often still being assessed according to traditional standards. However, we feel the incentive structure is quickly changing, as Open Science practices are increasingly being rewarded (Gernsbacher, 2018; Kidwell et al., 2016). PIs are in a more powerful situation than ECRs to implement Open Science on a broader level. If PIs who believe in Open Science actively fought for sustainable changes towards Open Science, adopting Open Science practices will no longer put ECRs at a disadvantage (for instance, for our current positions, experience with or interest in Open Science is considered a prerequisite). Second, we think Open Science is not an all-or-none commitment. For researchers who remain skeptical of certain aspects of Open Science, they can still incorporate other Open Science practices into their workflow. For instance, ECRs may be concerned that submitting their work to Open Access journals may lead to lower impact factors and jeopardize their careers (see below). In such cases, they may still try to publish in traditional closed high-profile journals, but make data and materials open, and share their manuscript via Green Open Access. We believe as more researchers adopt Open Science practices, the incentive structure for Open Science will be in place sooner.

As already mentioned, another issue concerns the benefits and drawbacks of Open Access publications. Because Diamond and Gold Open Access are our desired publication routes, we have to accept that we will not always attempt to publish in closed journals with high impact factors. Accordingly, this may result in more publications with lower impact factors compared to other ECRs. This could harm our careers, because the impact factors of one’s publications are still a major factor in the competition for research positions. However, we are convinced that Open Science will lead to higher research quality. Additionally, the citation advantage associated with Open Access publications could increase the impact of our work and our chances of finding a permanent position (McKiernan et al., 2016; Piwowar & Vision, 2013; Wang et al., 2015).

Moreover, in these times, the academic job market is very competitive. Therefore, we think it is important to have additional skills to stand out. In our view, experience with Open Science may become an essential skill for the job market (Working Group on Rewards under Open Science, 2017). Furthermore, many concrete skills that we will acquire when adopting Open Science practices, such as the use of version control systems, programming in popular open-source software such as Python, JavaScript, and R, will also benefit our careers. As with any investment, the potential benefits are greater the closer to the ground floor you get in. We believe this is an excellent time to gain Open Science experience. Having experience with Open Science is not only relevant from a practical perspective, that is, we know how to implement Open Science into our workflow; rather, and more importantly, having experience with Open Science says a lot about our understanding of how we want to work as scientists (Gewin, 2016). Doing Open Science as a whole lab will increase our visibility as Open Science practitioners and in turn raise the probability that we will be identified as such.

## Conclusion

To conclude, Open Science changes the way we do research. For us and probably many other researchers, doing science is more than a job. The principles of Open Science allow us to honor everything we love about science and to protect the quality of our work, so that we can be proud of it. We believe that Open Science brings researchers together so that they can revive what science is about at its core: striving to discover regularities about the world and helping to improve the way we live, sharing work with colleagues, as well as building up humanity’s knowledge (Grubaugh, 2017). In practice, we are aware that Open Science still has certain costs that can be daunting, especially to ECRs. A major concern is that the implementation of Open Science practices takes time at the beginning and may slow down the workflow, especially the publication process. However, we are convinced that it is time well spent. For us, the principles of Open Science not only represent good research practices, so that the quality of research will be improved; rather, the principles of Open Science liberate researchers and empower them to use a creative mindset when thinking about their work and designing new studies. In our lab, the time investment for Open Science is rewarded with meaningful co-authorships as part of the co-pilot system. Finally, we also hope to encourage ECRs to seek out PIs who actively support Open Science practices instead of compromising on their ideals (Allen & Mehler, 2019; Poldrack, 2019). Doing Open Science is easier when all lab members – and especially PIs – have an Open Science state of mind.

## Additional File

The additional file for this article can be found as follows:

10.5334/pb.494.s1 Appendix. Checklist for the Co-Pilot System.

## References

[1] Allen, C., & Mehler, D. M. (2019). Open Science challenges, benefits and tips in early career and beyond. PLoS Biology, 17(5): e3000246 DOI: 10.1371/journal.pbio.3000246 31042704 PMC6513108

[2] Arslan, R. C. (2019). How to automatically document data with the codebook package to facilitate data reuse. Advances in Methods and Practices in Psychological Science, 2(2), 169–187. DOI: 10.1177/2515245919838783

[3] Bakker, M., & Wicherts, J. M. (2011). The (mis)reporting of statistical results in psychology journals. Behavior Research Methods, 43(3), 666–678. DOI: 10.3758/s13428-011-0089-5 21494917 PMC3174372

[4] Brand, A., Allen, L., Altman, M., Hlava, M., & Scott, J. (2015). Beyond authorship: Attribution, contribution, collaboration, and credit. Learned Publishing, 28(2), 151–155. DOI: 10.1087/20150211

[5] Button, K. S., Ioannidis, J. P. A., Mokrysz, C., Nosek, B. A., Flint, J., Robinson, E. S. J., & Munafò, M. R. (2013). Power failure: Why small sample size undermines the reliability of neuroscience. Nature Reviews Neuroscience, 14(5), 365–376. DOI: 10.1038/nrn3475 23571845

[6] Camerer, C. F., Dreber, A., Holzmeister, F., Ho, T.-H., Huber, J., Johannesson, M., Wu, H., et al. (2018). Evaluating the replicability of social science experiments in Nature and Science between 2010 and 2015. Nature Human Behaviour, 2(9), 637–644. DOI: 10.1038/s41562-018-0399-z 31346273

[7] Chambers, C. D. (2013). Registered Reports: A new publishing initiative at Cortex. Cortex, 49(3), 609–610. DOI: 10.1016/j.cortex.2012.12.016 23347556

[8] cOAlition, S. (2019). Plan S. Retrieved from https://www.coalition-s.org/about/

[9] Colavizza, G., Hrynaszkiewicz, I., Staden, I., Whitaker, K., & McGillivray, B. (2019). The citation advantage of linking publications to research data. Retrieved from https://arxiv.org/abs/1907.02565 10.1371/journal.pone.0230416 PMC7176083 32320428

[10] De Leeuw, J. R. (2015). jsPsych: A JavaScript library for creating behavioral experiments in a Web browser. Behavior Research Methods, 47(1), 1–12. DOI: 10.3758/s13428-014-0458-y 24683129

[11] Donoho, D. L. (2010). An invitation to reproducible computational research. Biostatistics, 11(3), 385–388. DOI: 10.1093/biostatistics/kxq028 20538873

[12] Ebersole, C. R., Axt, J. R., & Nosek, B. A. (2016). Scientists’ reputations are based on getting it right, not being right. PLOS Biology, 14(5), e1002460 DOI: 10.1371/journal.pbio.1002460 27171138 PMC4865149

[13] Fanelli, D. (2018). Opinion: Is science really facing a reproducibility crisis, and do we need it to? Proceedings of the National Academy of Sciences, 115(11), 2628–2631. DOI: 10.1073/pnas.1708272114 PMC5856498 29531051

[14] Foster. (n.d.). Retrieved from https://www.fosteropenscience.eu/foster-taxonomy/open-science-definition

[15] Frankenhuis, W. E., & Nettle, D. (2018). Open Science is liberating and can foster creativity. Perspectives on Psychological Science, 13(4), 439–447. DOI: 10.1177/1745691618767878 29961408 PMC6041740

[16] Gernsbacher, M. A. (2018). Rewarding research transparency. Trends in Cognitive Sciences, 22(11), 953–956. DOI: 10.1016/j.tics.2018.07.002 30041865 PMC6195839

[17] Gewin, V. (2016). Data sharing: An open mind on open data. Nature, 529, 117–119. DOI: 10.1038/nj7584-117a 26744755

[18] Gilbert, D. T., King, G., Pettigrew, S., & Wilson, T. D. (2016). Comment on “Estimating the reproducibility of psychological science.” Science, 351(6277), 1037–1037. DOI: 10.1126/science.aad7243 26941311

[19] Giner-Sorolla, R. (2012). Science or Art? How aesthetic standards grease the way through the publication bottleneck but undermine science. Perspectives on Psychological Science, 7(6), 562–571. DOI: 10.1177/1745691612457576 26168113

[20] Grubaugh, N. (2017). Open science combats Zika. Retrieved from https://naturemicrobiologycommunity.nature.com/users/40355-nathan-grubaugh/posts/17015-open-science-combats-zika

[21] Hardwicke, T. E., & Ioannidis, J. (2018). Mapping the universe of registered reports. DOI: 10.31222/osf.io/fzpcy 31558810

[22] Hardwicke, T. E., Mathur, M. B., MacDonald, K. E., Nilsonne, G., Banks, G. C., Kidwell, M., Frank, M. C., et al. (2018). Data availability, reusability, and analytic reproducibility: Evaluating the impact of a mandatory open data policy at the journal Cognition. Royal Society Open Science, 5(8). DOI: 10.1098/rsos.180448 PMC6124055 30225032

[23] Houtkoop, B. L., Chambers, C., Macleod, M., Bishop, D. V. M., Nichols, T. E., & Wagenmakers, E.-J. (2018). Data Sharing in Psychology: A survey on barriers and preconditions. Advances in Methods and Practices in Psychological Science, 1(1), 70–85. DOI: 10.1177/2515245917751886

[24] Imming, M., & Tennant, J. (2018). DOI: 10.5281/zenodo.1285575

[25] Ioannidis, J. P. A. (2005). Why most published research findings are false. PLoS Medicine, 2(8), e124 DOI: 10.1371/journal.pmed.0020124 16060722 PMC1182327

[26] John, L. K., Loewenstein, G., & Prelec, D. (2012). Measuring the prevalence of questionable research practices with incentives for truth telling. Psychological Science, 23(5), 524–532. DOI: 10.1177/0956797611430953 22508865

[27] Johnson, V. E., Payne, R. D., Wang, T., Asher, A., & Mandal, S. (2017). On the reproducibility of psychological science. Journal of the American Statistical Association, 112(517), 1–10. DOI: 10.1080/01621459.2016.1240079 29861517 PMC5976261

[28] Kerr, N. L. (1998). HARKing: Hypothesizing After the Results are Known. Personality and Social Psychology Review, 2(3), 196–217. DOI: 10.1207/s15327957pspr0203_4 15647155

[29] Kidwell, M. C., Lazarević, L. B., Baranski, E., Hardwicke, T. E., Piechowski, S., Falkenberg, L.-S., Nosek, B. A., et al. (2016). Badges to acknowledge open practices: A simple, low-cost, effective method for increasing transparency. PLOS Biology, 14(5), e1002456 DOI: 10.1371/journal.pbio.1002456 27171007 PMC4865119

[30] Klein, O., Hardwicke, T. E., Aust, F., Breuer, J., Danielsson, H., Hofelich Mohr, A., Frank, M. C., et al. (2018). A practical guide for transparency in psychological science. Collabra: Psychology, 4(1), 20 DOI: 10.1525/collabra.158

[31] Klein, R. A., Ratliff, K. A., Vianello, M., Adams, R. B., Bahník, Š., Bernstein, M. J., Nosek, B. A., et al. (2014). Investigating Variation in Replicability: A “many labs” replication project. Social Psychology, 45(3), 142–152. DOI: 10.1027/1864-9335/a000178

[32] Leonelli, S. (2018). Re-thinking reproducibility as a criterion for research quality. Retrieved from http://philsci-archive.pitt.edu/id/eprint/14352 DOI: 10.1108/S0743-41542018000036B009

[33] Lewandowsky, S., & Bishop, D. (2016). Don’t let transparency damage science. Nature, 529(7587), 459–461. DOI: 10.1038/529459a 26819029

[34] McKiernan, E. C., Bourne, P. E., Brown, C. T., Buck, S., Kenall, A., Lin, J., Yarkoni, T., et al. (2016). How open science helps researchers succeed. ELife, 5, e16800 DOI: 10.7554/eLife.16800 27387362 PMC4973366

[35] Nosek, B. A., Ebersole, C. R., DeHaven, A. C., & Mellor, D. T. (2018). The preregistration revolution. Proceedings of the National Academy of Sciences, 115(11), 2600–2606. DOI: 10.1073/pnas.1708274114 PMC5856500 29531091

[36] Nuijten, M. B., Hartgerink, C. H. J., van Assen, M. A. L. M., Epskamp, S., & Wicherts, J. M. (2016). The prevalence of statistical reporting errors in psychology (1985–2013). Behavior Research Methods, 48(4), 1205–1226. DOI: 10.3758/s13428-015-0664-2 26497820 PMC5101263

[37] Nuzzo, R. (2015). How scientists fool themselves – and how they can stop. Nature, 526(7572), 182–185. DOI: 10.1038/526182a 26450039

[38] Open Access Library. (2018a). Diamond Open Access. Retrieved from https://oalibrary.org/diamond-open-access/

[39] Open Access Library. (2018b). Gold Open Access. Retrieved from https://oalibrary.org/gold-open-access/

[40] Open Science Collaboration. (2015). Estimating the reproducibility of psychological science. Science, 349(6251), aac4716 DOI: 10.1126/science.aac4716 26315443

[41] Pashler, H., & Wagenmakers, E. (2012). Editors’ Introduction to the Special Section on Replicability in Psychological Science: A crisis of confidence? Perspectives on Psychological Science, 7(6), 528–530. DOI: 10.1177/1745691612465253 26168108

[42] Patil, P., Peng, R. D., & Leek, J. T. (2016). A statistical definition for reproducibility and replicability. BioRxiv. DOI: 10.1101/066803

[43] Peirce, J. W. (2007). PsychoPy—Psychophysics software in Python. Journal of Neuroscience Methods, 162(1–2), 8–13. DOI: 10.1016/j.jneumeth.2006.11.017 17254636 PMC2018741

[44] Piwowar, H. A., & Vision, T. J. (2013). Data reuse and the open data citation advantage. PeerJ, 1, e175 DOI: 10.7717/peerj.175 24109559 PMC3792178

[45] Poldrack, R. A. (2019). The costs of reproducibility. Neuron, 101(1), 11–14. DOI: 10.1016/j.neuron.2018.11.030 30605654

[46] R Core Team. (2013). R: A language and environment for statistical computing. Retrieved from http://www.R-project.org/

[47] Reason, J. (2000). Human error: models and management. BMJ, 320(7237), 768–770. DOI: 10.1136/bmj.320.7237.768 10720363 PMC1117770

[48] Simmons, J. P., Nelson, L. D., & Simonsohn, U. (2011). False-Positive Psychology: Undisclosed flexibility in data collection and analysis allows presenting anything as significant. Psychological Science, 22(11), 1359–1366. DOI: 10.1177/0956797611417632 22006061

[49] Steegen, S., Tuerlinckx, F., Gelman, A., & Vanpaemel, W. (2016). Increasing transparency through a multiverse analysis. Perspectives on Psychological Science, 11(5), 702–712. DOI: 10.1177/1745691616658637 27694465

[50] Stodden, V., Seiler, J., & Ma, Z. (2018). An empirical analysis of journal policy effectiveness for computational reproducibility. Proceedings of the National Academy of Sciences, 115(11), 2584–2589. DOI: 10.1073/pnas.1708290115 PMC5856507 29531050

[51] Stroebe, W., & Strack, F. (2014). The alleged crisis and the illusion of exact replication. Perspectives on Psychological Science, 9(1), 59–71. DOI: 10.1177/1745691613514450 26173241

[52] Suber, P. (2012). Open Access. Cambridge, Massachusetts: MIT Press.

[53] The Slow Science Academy. (2010). The Slow Science Manifesto. Retrieved from http://slow-science.org/

[54] van ’t Veer, A. E., & Giner-Sorolla, R. (2016). Pre-registration in social psychology. A discussion and suggested template. Journal of Experimental Social Psychology, 67, 2–12. DOI: 10.1016/j.jesp.2016.03.004

[55] Veldkamp, C. L. S., Nuijten, M. B., Dominguez-Alvarez, L., van Assen, M. A. L. M., & Wicherts, J. M. (2014). Statistical reporting errors and collaboration on statistical analyses in psychological science. PLoS ONE, 9(12), e114876 DOI: 10.1371/journal.pone.0114876 25493918 PMC4262438

[56] Wang, X., Liu, C., Mao, W., & Fang, Z. (2015). The open access advantage considering citation, article usage and social media attention. Scientometrics, 103(2), 555–564. DOI: 10.1007/s11192-015-1547-0

[57] Washburn, A. N., Hanson, B. E., Motyl, M., Skitka, L. J., Yantis, C., Wong, K. M., Carsel, T. S., et al. (2018). Why do some psychology researchers resist adopting proposed reforms to research practices? A description of researchers’ rationales. Advances in Methods and Practices in Psychological Science, 1(2), 166–173. DOI: 10.1177/2515245918757427

[58] Wessel, J. R., Gorgolewski, K. J., & Bellec, P. (2019). Switching software in science: Motivations, challenges, and solutions. DOI: 10.1016/j.tics.2019.01.004 30712996

[59] Wicherts, J. M. (2011). Psychology must learn a lesson from fraud case. Nature, 480(7375), 7 DOI: 10.1038/480007a 22129686

[60] Wiegmann, D. A., & Shappell, S. A. (2003). A human error approach to aviation accident analysis: The human factors analysis and classification system. Aldershot, Hants, England; Burlington, VT: Ashgate.

[61] Wilkinson, M. D., Dumontier, M., Aalbersberg, I. J., Appleton, G., Axton, M., Baak, A., Mons, B., et al. (2016). The FAIR Guiding Principles for scientific data management and stewardship. Scientific Data, 3, 160018 DOI: 10.1038/sdata.2016.18 26978244 PMC4792175

[62] Working Group on Rewards under Open Science. (2017, 7). Evaluation of Research Careers fully acknowledging Open Science Practices. Retrieved from https://ec.europa.eu/research/openscience/pdf/os_rewards_wgreport_final.pdf

