# Supplementary material for: Open Up – the Mission Statement of the Control of Impulsive Action (Ctrl-ImpAct) Lab on Open Science
Source: Psychol Belg. 2019 Aug 20;59(1):321–37. doi: 10.5334/pb.494 (PMC6707000; doi:10.5334/pb.494)
Supplement: Appendix. — Checklist for the Co-Pilot System. [file pb-59-1-494-s1.pdf]

Appendix

Checklist for the Co-Pilot System

	P	CP	Remarks
Study Preparation & Hypotheses			
Create GitHub repository for co-piloting[1]			
Write a data management plan[2]			
Prepare experimental materials (code etc.)			
Write data documentation file			
Pre-register on OSF (pre-registration & materials)			
Data Collection & Analysis			
Analyze data and document analysis steps			
Prepare manuscript/short report with results			
Compare the report with the pre-registration			
Upload lab notes, anonymized raw data and analysis files to OSF			
Manuscript & Communication			
Submit to an Open Access journal			
Upload the manuscript to a preprint server			
Make the OSF projects public			

Notes: P: pilot researcher; CP: co-pilot researcher

Links:

1. https://github.com/fredvbrug/Ctrl-ImpAct-copilot-guidelines
2. https://github.com/fredvbrug/Data-management-guidelines
